# The Naturally Occurring YMDD Mutation among Patients Chronically Infected HBV and Untreated with Lamivudine: A Systematic Review and Meta-Analysis

**DOI:** 10.1371/journal.pone.0032789

**Published:** 2012-03-27

**Authors:** Youwen Tan, Keqin Ding, Jing Su, Xuan Trinh, Zhihang Peng, Yuhua Gong, Li Chen, Qian Cui, Na Lei, Xin Chen, Rongbin Yu

**Affiliations:** 1 Department of Epidemiology and Biostatistics, School of Public Health, Nanjing Medical University, Nanjing, China; 2 Department of Hepatosis, The Third People's Hospital of Zhenjiang City, Zhenjiang, China; 3 Department of Pharmacology, University of Gothenburg, Göteborg, Sweden; University Hospital of Essen, Germany

## Abstract

**Background:**

Several recent reports have demonstrated that tyrosine (Y)-methionine (M)-aspartic acid (D)-aspartic acid (D) (YMDD) motif mutations can naturally occur in chronic HBV patients without antiviral treatment such as lamivudine therapy. This paper aims to assess the overall spontaneous incidence and related risk factors of YMDD-motif mutations among lamivudine-naïve chronic HBV carriers, so as to provide some clue for clinical treatment of hepatitis B.

**Methodology/Principal Findings:**

Chinese and English literatures were searched for studies reporting natural YMDD mutations among untreated chronic HBV patients from 2001 to 2010. The incidence estimates were summarized and analyzed by meta-analyses. Forty-seven eligible articles from eight countries were selected in this review (13 in English and 34 in Chinese). The pooled incidence of YMDD-motif mutation among untreated chronic HBV patients from eight countries was 12.21% (95% CI: 9.69%–14.95%). China had an incidence of 13.38% (95% CI: 10.90%–16.07%) and seven other countries had an incidence of 9.90% (95% CI: 3.28%–19.55%), respectively. Lamivudine therapy would increase the risk of mutations 5.23 times higher than the untreated patients. A higher HBV DNA copy number was associated with increased incidence of natural YMDD mutation. No significant difference was found in YMDD mutation incidence between groups of different gender, age, HBeAg status, patients' ALT (alanine aminotransferase) level, and between the groups of HBV genotype B and C.

**Conclusions:**

The YMDD-motif mutations can occur spontaneously with a relatively high incidence in CHB patients untreated with lamivudine. These mutations might be the consequence of accumulated base mismatch due to the nature of viral polymerase. More fundamental and clinical studies are needed to clarify the influence of YMDD mutations in hepatitis B progression and antiviral treatment.

## Introduction

Hepatitis B virus (HBV) infection has long been a serious public health problem around the world. HBV is one of the main causes of acute and chronic hepatitis in humans. Nearly 400 million people are infected chronically with HBV worldwide [1]. It has been demonstrated that chronic infection with this virus is linked to the development of cirrhosis and hepatocellular carcinoma, accounting for 0.5–0.75 million deaths per year.

Antiretroviral treatment is the main clinical treatment of chronic hepatitis B (CHB). Lamivudine, a nucleoside analogue, has been widely used because of its high effectiveness. However, long-term use of lamivudine may lead to the emergence of lamivudine-resistance in some HBV cases. The resistance generation is closely associated with mutations in the highly conserved YMDD motif, which is in the catalytic domain C of viral DNA polymerase. The YMDD motif has an amino acid sequence of tyrosine (Y)-methionine (M)-aspartic acid (D)-aspartic acid (D) [2] and is both the binding and functional site of lamivudine.

Recent reports revealed that besides the mutations caused by lamivudine therapy, some YMDD mutations can occur spontaneously in lamivudine-untreated CHB patients. Because HBV reverse transcriptase has no proofreading activity during the replication process, mutations can naturally occur due to random nucleotide misincorporation [3]. The most common substitutions are methionine at amino acid position-204 to either isoleucine (rtM204I, YIDD mutant) or valine (rtM204V, YVDD mutant) [4], [5]. Many studies have reported the incidence and characteristic of spontaneous YMDD-motif mutation in lamivudine-naive CHB patients. However, the reported incidences were quite different (0% to 31.58%) or even contradict with each other. This paper reviews the available publications, aiming to comprehensively provide not only the incidence but also associated factors of natural YMDD-motif mutation among lamivudine-untreated CHB patients. The results should provide scientific evidences for clinical treatment and future research of YMDD mutation.

## Materials and Methods

### Search strategy

A number of sources were used to access data for this review. Published articles were searched in PubMed (http://www.ncbi.nlm.nih.gov/pubmed) and Embase (http://www.embase.com/search) database from January 2001 to December 2010 using the search terms: YMDD, lamivudine, HBV OR hepatitis B, spontaneous mutation OR natural mutation, untreated, and naive. The two Chinese databases: China National Knowledge Infrastructure (CNKI) (http://epub.edu.cnki.net/) and WanFang (http://g.wanfangdata.com.cn/) were also searched for articles related to HBV and YMDD motif mutation from January 2001 to December 2010. The reference lists of relevant articles were also searched manually for complement. This review was conducted and reported according to the PRISMA (Preferred Reporting Items for Systematic Reviews and Meta-Analyses) Statement issued in 2009 (Table S1) [6].

### Selection criteria

All the potentially relevant papers were reviewed independently by two investigators through assessing the eligibility of each article and useful data were extracted with standardized data-extraction forms. Studies published in English and Chinese were eligible if they fulfilled the following criteria: (1) studies in the mentioned four databases with full text, or with available data in abstract; (2) study objects contain CHB patients with NO treatment of lamivudine or other antiviral drugs; (3) studies report the rates of YMDD mutation among lamivudine-untreated CHB cases.

The exclusion criteria were: (1) studies without specific sample origins; (2) studies with overlapping time intervals of the same sample collection; (3) study objects were treated with lamivudine; (4) study objects were patients with acute hepatitis B; (5) subset of a published article by the same authors; (6) studies present unclear data or obviously paradoxical data.

### Data extraction and quality assessment

The following information, though some studies did not contain all of them, were extracted from the literatures: first author's name, publication date, journal title, study area, sample size (the number of CHB patients untreated with lamivudine), the number of cases with YMDD mutation, the method of detecting YMDD mutation, gender and average age of study objects, the status of HBeAg (positive or negative), the amount of serum HBV DNA, ALT (alanine aminotransferase) level, and HBV genotype. All the numerical values of HBV DNA copies were converted to logarithm. Two investigators (Keqin Ding and Yuhua Gong) extracted and collected data independently using a standardized data-extraction protocol. Any discrepancy was settled by consensus of the whole team.

### Statistical analysis

In this study, random effect model or fixed effect model was used for meta-analysis, according to the heterogeneity between studies which was tested with the Q test (*P*<0.10 was considered indicative of statistically significant heterogeneity) and the *I^2^* statistic (values of 25%, 50% and 75% are considered to represent low, medium and high heterogeneity respectively). The fixed effect model was used when there was no significant heterogeneity (*I^2^*<50%); otherwise the random effect model was used. P values were calculated by χ^2^ tests. All the reported P values are two-sided and P values less than 0.05 were regarded as statistically significant for all included studies.

Publication bias was examined by a funnel plot of log OR (odds ratio) against its standard error using Begg's test, and the degree of asymmetry was tested statistically using Egger's unweighed regression asymmetry test. Publication bias exists if the rank correlation coefficient from Begg's test is close to 1, or the slope parameter from Egger regression is high.

Data manipulation and statistical analyses were undertaken using the Statistical Software Package (STATA) 10.0 (STATA Corporation, College Station, TX, USA, 2009) and Review Manager 4.2 (The Cochrane Collaboration, Oxford, United Kingdom, 2003).

## Results

**Figure 1:**
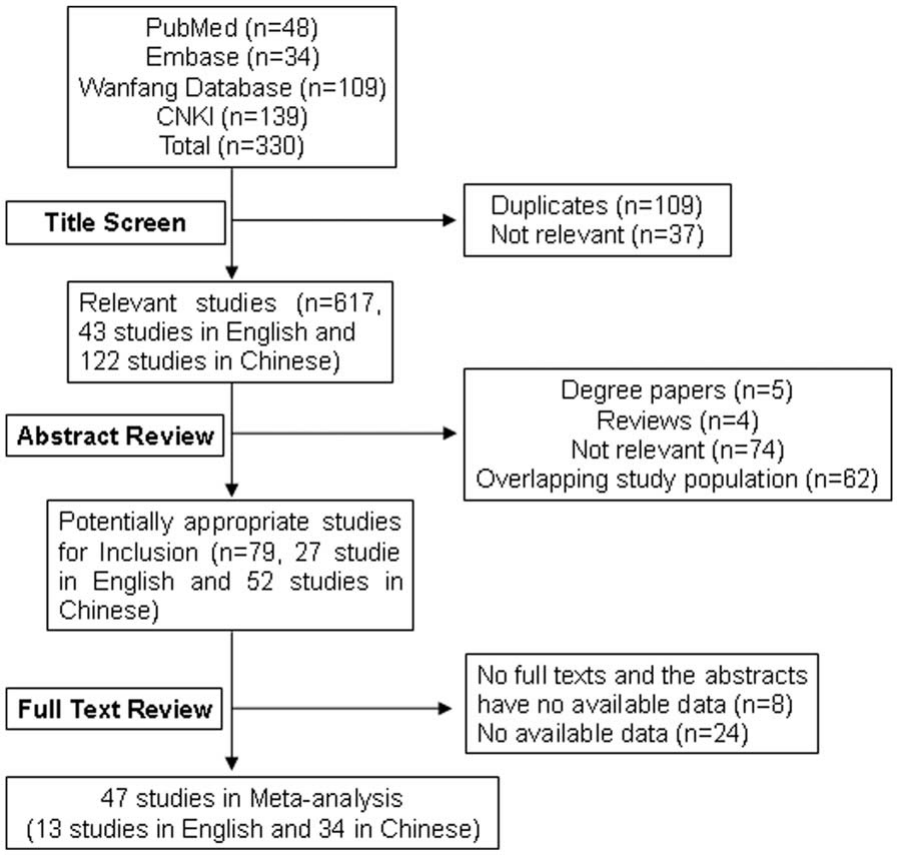
Flow diagram of the systematic literature research.

According to the literature search strategies, a total of 202 studies (54 studies in PubMed and Embase, and 148 studies in CNKI and Wanfang database) were identified, and 155 studies were excluded based on the inclusion and exclusion criteria. Finally there were 47 studies adopted in this study (13 studies in English and 34 studies in Chinese) as showed in Figure 1 and Figure S1 [6].

The studies of our review were involved in the following 18 provinces and municipalities in China and seven other countries: Central China (Hubei, Hunan), North China (Beijing, Liaoning, Jilin, Shan'xi), South China(Hainan, Guangdong, Guangxi), East China (Shanghai, Shandong, Jiangsu, Zhejiang), Northwest China (Shaanxi, Xinjiang), and Southwest China (Chongqing, Sichuan, Yunnan); Japan, Korea, Iran, Italy, Turkey, Jordan, and South Africa. A total of 4,555 CHB patients untreated with lamivudine and other antiviral drugs were involved in this review. A great majority of these cases were collected from hospitals.

### Difference in the incidence of YMDD mutation between lamivudine-treated cases and lamivudine-untreated cases

**Figure 2:**
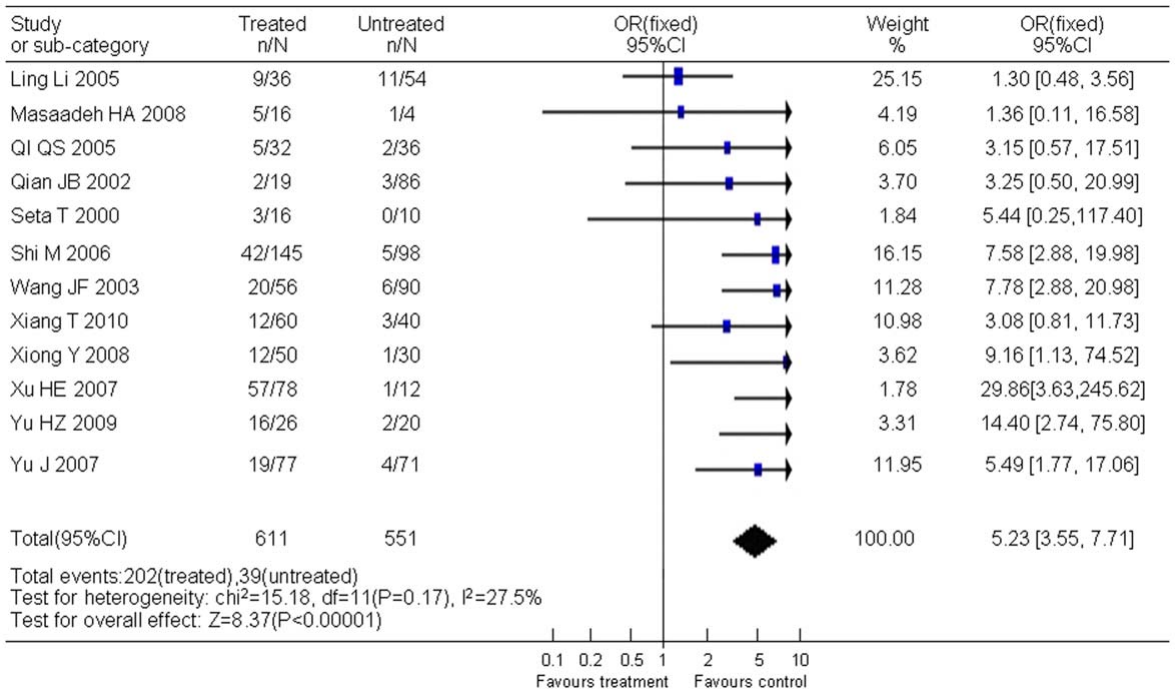
Difference in the incidence of YMDD mutation between lamivudine-treated patients and untreated patients.

Twelve studies compared YMDD mutation incidence between 611 lamivudine-treated CHB patients and 551 untreated CHB patients. There was no significant statistical heterogeneity (χ^2^ = 15.18, *P* = 0.17, *I^2^* = 27.5%). The meta-analysis with fixed effects models demonstrated higher YMDD mutation rate in lamivudine-treated CHB cases than in untreated CHB cases (OR = 5.23, 95% CI: 3.55–7.71; Z = 8.37, *P*<0. 01) (Figure 2). Begg's test and Egger's test showed that publication bias existed.

### Incidence of YMDD motif mutation among lamivudine-untreated CHB patients

As showed in Table 1, the pooled incidence of all the eight countries in YMDD mutation among lamivudine-untreated cases is 12.21% (95% CI: 9.69%–14.95%), while the pooled incidence in China is 13.38% (95% CI: 10.90%–16.07%). We also calculated the incidence in each sub-region of China. The naturally occurring YMDD mutation was relatively more severe in Northwest China and Southwest China, where the incidences were 20.19% (95% CI: 14.03%–27.10%) and 17.23% (95% CI: 6.07%–32.58%), respectively. The lowest incidence was in north China with the rate of 6.71% (95% CI: 3.87%–10.26%). The incidences of naturally occurring YMDD motif mutation in Central China, East China, and South China were 12.77% (95% CI: 7.06%–19.87%), 11.18% (95% CI: 8.34%–14.35%), and 14.91% (95% CI: 8.04%– 23.41%), respectively. In addition, the pooled incidence of the seven foreign countries was 9.90% (95% CI: 3.28%–19.55%).

**Table 1:** Pooled incidence of YMDD mutation among CHB patients untreated with antiviral drugs in each region.

Region		Province	Author	Year	Untreated patients	Cases of YMDD mutation	Incidence of YMDD mutation (95%CI )(%)	Pooled incidence (95%CI)(%)	Weight (%)
**China**	**Central China**	**Hubei**	Xiang T [7]	2010	40	3	8.51 ( 2.02, 18.84)	12.77 ( 7.06, 19.87)	7.10
			Wei J [8]	2010	449	46	10.35 ( 7.69, 13.31)		
		**Hunan**	Tang XL [9]	2009	119	23	19.59 ( 13.01, 27.14)		
	**North China**	**Liaoning**	Shi M [10]	2006	98	5	5.54 ( 1.92, 10.87)	6.71 ( 3.87, 10.26)	8.34
		**Shan'xi**	Qi QS [11]	2005	36	2	6.68 ( 1.01, 16.85)		
		**Jilin**	Xiong Y [12]	2008	30	1	4.70 ( 0.18, 14.77)		
		**Beijing**	Ren YQ [13]	2004	65	6	9.84 ( 3.89, 18.10)		
	**East China**	**Shandong**	Wu C [14]	2007	38	3	8.93 ( 2.13, 19.75)	11.18 ( 8.34, 14.35)	30.30
		**Shanghai**	Wang JF [15]	2003	90	6	7.14 ( 2.78, 13.28)		
			Min XC [16]	2009	196	21	10.90 ( 6.96, 15.63)		
			Huo H [17]	2006	183	40	22.02 ( 16.33, 28.26)		
		**Jiangsu**	Yu HZ [18]	2009	20	2	11.79 ( 1.87, 28.62)		
			Qian JB [19]	2002	86	3	4.01 ( 0.93, 9.10)		
			Qiu YW [20]	2008	380	63	16.66 ( 13.11, 20.55)		
			Cheng NL [21]	2008	170	14	8.48 ( 4.78, 13.11)		
		**Zhejiang**	Zhang JM [22]	2004	122	12	10.17 ( 5.47, 16.11)		
			Zheng JM [23]	2003	40	3	8.51 ( 2.02, 18.84)		
			Cheng FJ [24]	2006	89	12	13.90 ( 7.56, 21.74)		
			Zheng NH [25]	2007	229	16	7.17 ( 4.20, 10.84)		
			Yan MH [26]	2003	110	19	17.57 ( 11.09, 25.16)		
	**South China**	**Hainan**	WU J [27]	2009	49	7	14.99 ( 6.56, 26.08)	14.91 ( 8.04, 23.41)	16.03
		**Guangxi**	Huang ZM [28]	2005	104	28	27.14 ( 19.12, 36.00)		
			Qian YQ [29]	2005	125	30	24.22 ( 17.15, 32.02)		
		**Guangdong**	QU YY [30]	2006	140	20	14.52 ( 9.22, 20.79)		
			Liu jH [31]	2008	176	7	4.22 ( 1.77, 7.69)		
			Guo HB [32]	2004	29	6	21.65 ( 9.02, 37.88)		
			Yu J [33]	2007	71	4	6.24 ( 1.87, 12.94)		
	**NorthWest**	**Xinjiang**	Li CM [34]	2003	39	3	8.71 ( 2.07, 19.27)	20.19 ( 14.03, 27.10)	13.80
			Zhang XH [35]	2003	150	32	21.53 ( 15.34, 28.40)		
		**Shaanxi**	Qu JX [36]	2010	82	21	25.91 ( 17.11, 35.81)		
			Qu JX [37]	2008	106	28	26.65 ( 18.72, 35.38)		
			Pan HQ [38]	2010	110	31	28.40 (20.43, 37.11)		
			Zhang Y [39]	2005	60	5	8.99 ( 3.17, 17.38)		
	**SouthWest**	**Chongqing**	Li L [40]	2005	54	11	20.92 ( 11.28, 32.54)	17.23 ( 6.07, 32.58)	8.06
			Liu TH [41]	2004	55	3	6.22 ( 1.46, 13.97)		
		**Sichuan**	He X [42]	2007	12	1	11.25 ( 0.49, 33.19)		
		**Yunnan**	Wang YQ [43]	2009	190	60	31.70 ( 25.30, 38.42)		
	**Pooled incidence of YMDD mutation in China**							**13.38 ( 10.90, 16.07)**	83.63
**Other countries**	**Japan**		Matsuda M [44]	2004	20	1	6.96 ( 0.28, 21.36)	9.90 ( 3.28, 19.55)	16.37
			Seta T [45]	2000	10	0	2.32 ( 2.02, 18.80)		
			Kobayashi S [46]	2001	18	5	28.89 (11.31, 50.71)		
	**Korea**		Heo J [47]	2004	40	3	8.51 ( 2.01, 18.84)		
	**Iran**		AB-Olyaee S [48]	2008	147	0	0.17 ( 0.16, 1.47)		
			Ramezani A [49]	2008	77	0	0.32 ( 0.29, 2.78)		
	**Italy**		Villa E [50]	2009	11	0	2.13 ( 1.85, 17.34)		
	**Turkey**		Akarsu M [51]	2006	71	13	18.76 ( 10.65, 28.53)		
	**Jordan**		Masaadeh HA[52]	2008	4	1	29.48 ( 1.84, 71.94)		
	**South Africa**		Selabe SG [53]	2007	15	3	21.78 ( 5.67, 44.52)		
	**Pooled incidence of YMDD mutation in these eight countries**							**12.21 ( 9.69, 14.95)**	100.00

### Incidence of natural YMDD mutation between gender and age

**Figure 3:**
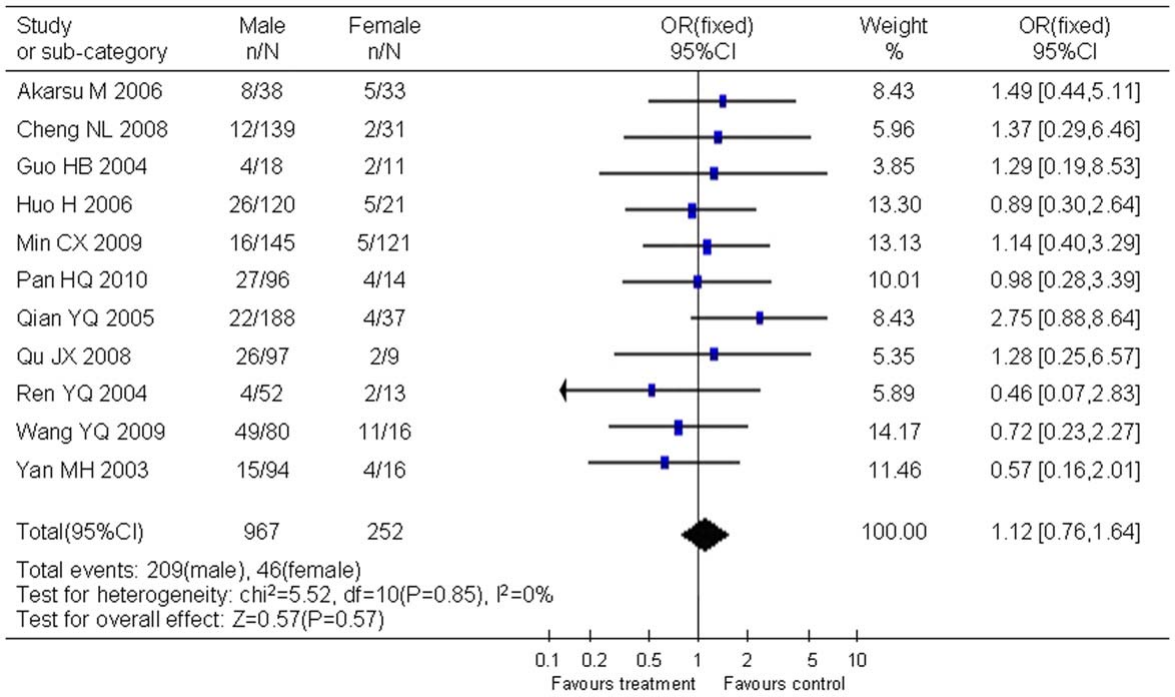
Difference in the incidence of YMDD mutation between male and female patients.

**Figure 4:**
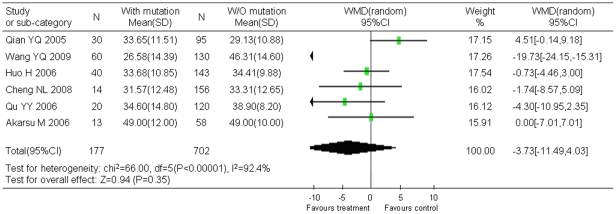
Difference in average ages between the patients with YMDD mutations and the patients without mutation.

Stratified analyses showed that natural YMDD mutation was not associated with gender (Figure 3) and age (Figure 4).

Eleven studies contained the available information of gender distribution, including 967 males and 252 females. There was no significant statistical heterogeneity (χ^2^ = 5.52, *P* = 0.85, *I^2^* = 0). Males and females had equal chances of natural YMDD mutation (OR = 1.12, 95% CI: 0.76–1.64; Z = 0.57, *P* = 0.57) (Figure 3). No significant publication bias was observed in this analysis.

The average age was mentioned in six studies including 177 cases with YMDD mutations and 702 cases without YMDD mutation. Statistical heterogeneity was observed between groups (χ^2^ = 66.00, *P*<0.00001, *I^2^* = 92.4%). By using the random effects models, no significant difference of average age was showed between groups (WMD (weighted mean difference) = −3.73, 95% CI: −11.49–4.03; Z = 0.94, *P* = 0.35) (Figure 4). Begg's test and Egger's test indicated that publication bias existed.

### Relation of natural YMDD mutation with pathogen-related characteristics

**Figure 5:**
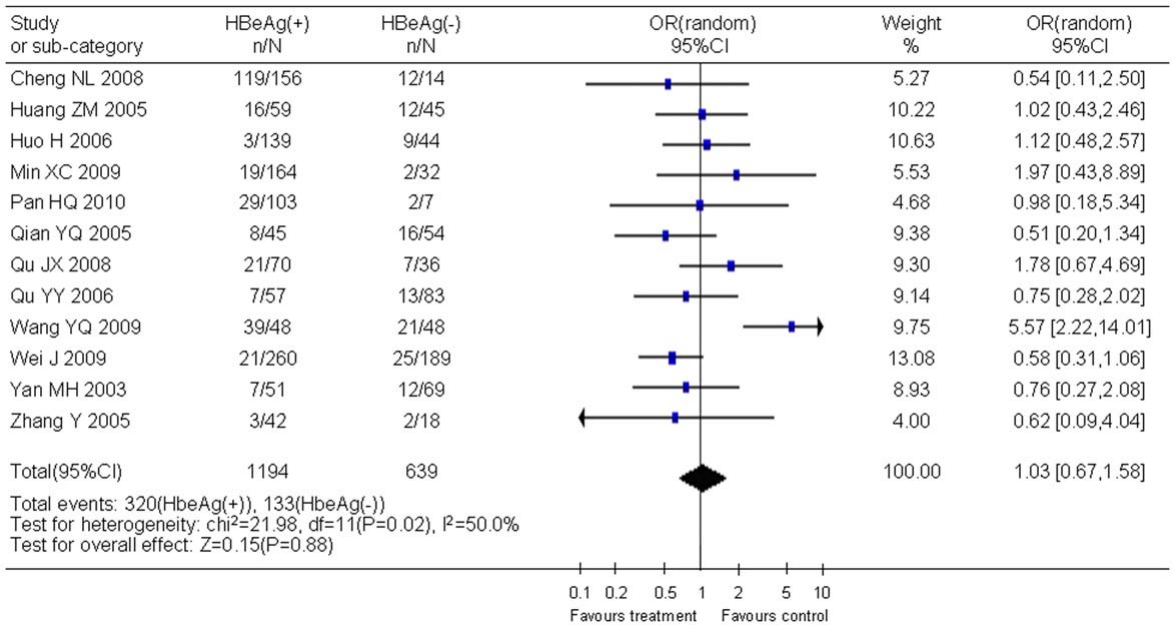
Difference in the incidence of YMDD mutation between HBeAg positive and HBeAg negative patients.

**Figure 6:**
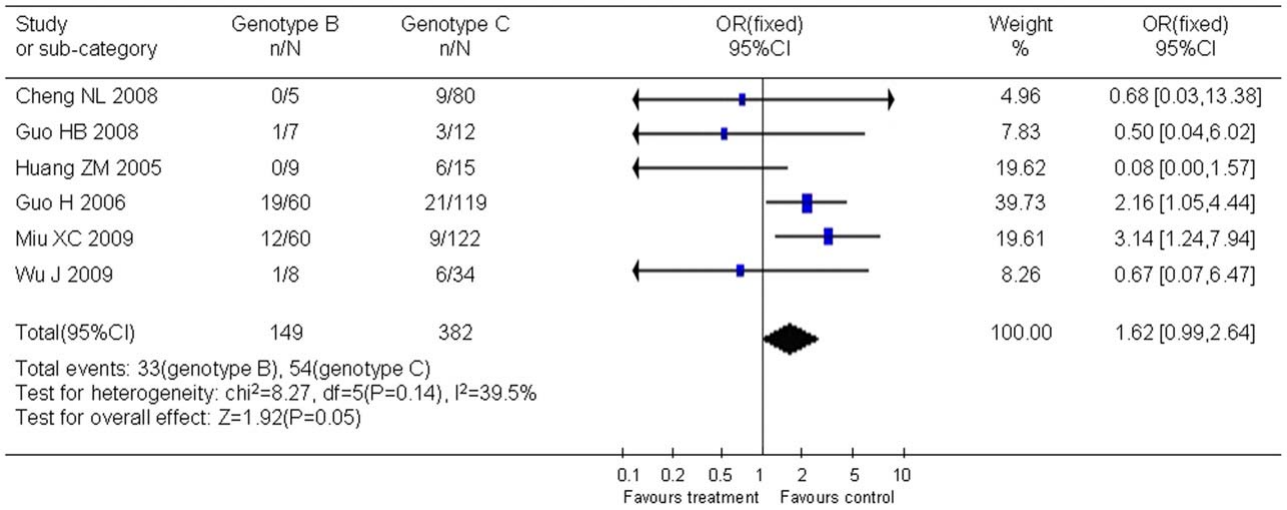
Difference in the incidence of YMDD mutation between patients with HBV genotype B and genotype C.

**Figure 7:**
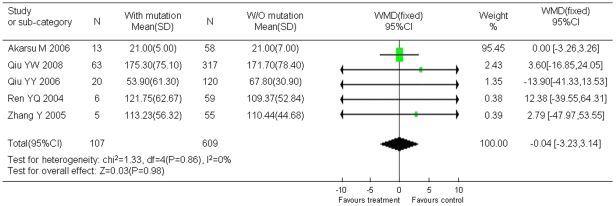
Difference in ALT level between the patients with YMDD mutations and the patients without mutation.

Statistical analyses also showed that the occurrence of YMDD mutation was not associated with HBeAg status (Figure 5), HBV genotype (Figure 6), and ALT level (Figure 7). However, viral load was showed to be associated with YMDD mutation. Neither statistical heterogeneity nor publication bias was observed in those analyses, except that publication bias existed in articles of HBV genotype analysis.

Twelve studies including 1194 HBeAg-positive cases and 639 HBeAg-negative cases showed that the incidence of natural YMDD-motif mutation was the same in different HBeAg status groups (OR = 1.03, 95% CI: 0.67–1.58; Z = 0.15, *P* = 0.88). It also seems that ALT level did not play a role in occurrence of natural YMDD mutation among patients with or without lamivudine therapy (WMD = −0.04, 95% CI: −3.23–3.14; Z = 0.03, *P* = 0.98). Six studies reported 149 genotype-B HBV cases and 382 genotype-C HBV cases. No significant difference was found in rates of natural YMDD mutation between genotype B and genotype C (OR = 1.62, 95% CI: 0.99–2.64; Z = 1.92, *P* = 0.05).

**Figure 8:**
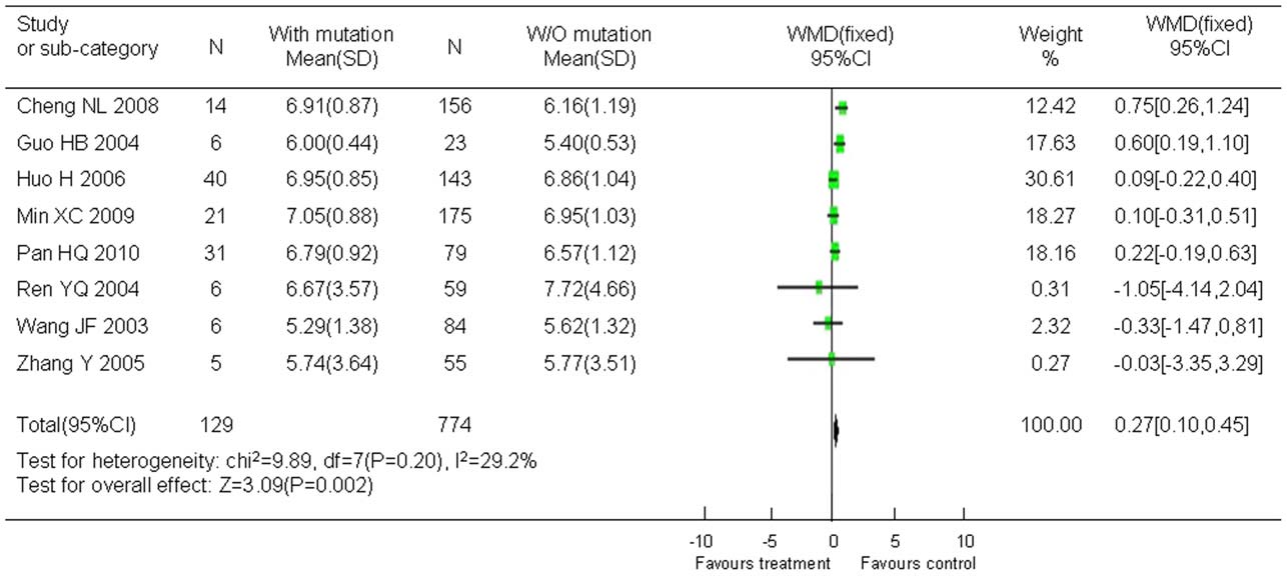
Difference in HBV DNA copies between the patients with YMDD mutations and the patients without mutation.

Eight studies (including 129 mutants and 774 cases without mutation) referred to the relation of HBV-DNA viral load with YMDD mutation. As showed in Figure 8, the HBV DNA copy number was higher in the group with YMDD mutation than in the group without mutation (WMD = 0.27, 95% CI: 0.10–0.45; Z = 3.09, P = 0.002).

## Discussion

The application of lamivudine is a breakthrough in viral treatment. Most patients with chronic hepatitis B had excellent response to lamivudine, but development of YMDD mutants attenuated efficacy of lamivudine treatment. Based on the studies included in this analysis, lamivudine treatment increased the risk of YMDD mutation to 5 fold of untreated controls (OR = 5.23, 95% CI: 3.55–7.71). Although mutations in YMDD motif increased during lamivudine treatment, many studies reported that this mutation could also be spontaneous. As showed in this report, the pooled incidence of natural YMDD mutation was as high as 12.21% (95% CI: 9.69%–14.95%). This result mostly represented the current situation in East Asia, especially China, since in this research 37 studies were from China and only 10 were from other 7 countries including Japan and Korea. It is not strange because China has a high prevalence of HBV and numbers of reports have been accumulated with clinical observation of YMDD mutations. However, the incidence of spontaneous YMDD mutations did not differ among countries (Table 1). Gender and age were not related with spontaneous YMDD mutations either (Figure 3 or Figure 4). These results suggest that pathogen factors rather than host genetic factors are the determinants of these natural YMDD mutants.

Of all the pathogen-related factors studied in this review, viral load was correlated with the incidence rate of natural YMDD mutation, while patients' HBeAg status and ALT level had no influence. A cohort study by Atkins M et al. reported that mutant virus strains were more likely to appear in lamivudine-treated patients with larger virus amount and longer therapy duration [54]. It seems like the YMDD mutation might occur at certain rate and accumulate during HBV replication. The relation between YMDD mutation and infection duration was not conclusive because it is difficult to determine the accurate infection duration. It is also hard to define a proper study interval. Min XC et al [16] found no difference in YMDD natural mutation between the group with 30-year or more infection duration and the group with less than 30-year duration. The issue is that 30-year duration may not be an appropriate study interval to detect accumulation effect of mutations.

Hepatitis B virus has eight genotypes classified from A to H, which variant in geographic distribution and characteristics. Genotypes B and C are predominant in East Asia such as China, Korea, and Japan [55]. A few reports showed an association between emergence rate of YMDD mutants and HBV genotypes. Huang ZM et al [28] demonstrated that the mutations mostly occur in genotype C and its mixed genotypes. Controversially Min XC et al [16] and Huo H et al [17] indicated these mutants emerge more frequently in genotype B virus than in genotype C virus. By this meta-analysis, no significant difference was observed in incidence of YMDD mutation between genotype B and genotype C. Due to inadequate information in the adopted literatures we failed to analyze the other genotypes in this review. Multinational studies would be valuable to clarify the relationship between the occurrence of YMDD mutants and HBV genotypes.

It has been commonly recognized that YMDD mutations can occur spontaneously. There are two major concerns following the finding. Firstly, do naturally occurring YMDD mutants influence the nature course of hepatitis B and worsen patients' condition? Secondly, since lamivudine treatment increases YMDD mutations, is efficacy of lamivudine reduced during treatment? Currently there is no evidence that YMDD mutations are associated with ALT level. The result indicates that at least naturally existing YMDD mutants may not worsen patients' liver function. Alien MI et al [4] demonstrated that the sensitivity of YMDD mutant strains to lamivudine declined and viral titers rebounded during lamivudine treatment *in vitro*. This finding does not mean necessarily that patients with pre-existing mutations have to be identified for special care or other therapy, because YMDD mutants were found to be attenuated in replication capacity and pathogenicity [44], [46]. In that case, YMDD mutants have little possibility to be dominant during infection and lamivudine should still be effective to treat hepatitis. All these opinions are not convincing enough to manipulate strategy of current lamivudine treatment and more fundamental and clinical evidences are needed to reveal the ultimate impact of YMDD mutations.

Considering the YMDD mutations could be acquired from other people instead of occur spontaneously, the natural mutation rate might be overestimated. However, there has been no laboratory evidence clarifying this possibility. Qu JX et al [36] indicated that children had higher mutation prevalence if YMDD mutants were detected in their parents. The question is that whether these mutants are transmitted from parents or genetic background increases mutation rate. Plus the conclusion was drawn from fourteen families and might just be a coincidence. Further studies are needed to distinguish acquired mutation from spontaneous mutation, including comparison of infectivity between the wild type and mutant virus strains, transfection assay of cells, and animal experiments.

In conclusion, YMDD-motif mutation can occur naturally with a rather high rate about 12.21% among untreated CHB patients. Patients' viral load is the factor that is admitted by majority to be correlated with mutation rate. Further mechanism researches are required to clarify the behaviors of mutants in addition to attenuated replication ability. Large-scale population studies in multi countries are also necessary to evaluate the influence of YMDD mutations in hepatitis B progression and antiviral treatment.

## Supporting Information

Figure S1 **PRISMA Flowchart.**
(DOC)

Table S1 **PRISMA Checklist.**
(DOC)
