# Supplementary material for: The Naturally Occurring YMDD Mutation among Patients Chronically Infected HBV and Untreated with Lamivudine: A Systematic Review and Meta-Analysis
Source: PLoS One. 2012 Mar 27;7(3):e32789. doi: 10.1371/journal.pone.0032789 (PMC3314000; doi:10.1371/journal.pone.0032789)
Supplement: Figure S1 — PRISMA Flowchart. (DOC) [file pone.0032789.s001.doc]

PubMed (n=48)

Embase (n=34)

Wanfang Database (n=109)

CNKI (n=139)

Total (n=330)

Duplicates (n=109)

Not relevant (n=37)

**Title Screen**

Relevant studies (n=617, 43 studies in English and

122 studies in Chinese)

**Abstract Review**

Degree papers (n=5)

Reviews (n=4)

Not relevant (n=74)

Overlapping study population (n=62)

Potentially appropriate studies for Inclusion (n=79, 27 studie in English and 52 studies in Chinese)

**Full Text Review**

No full texts and the abstracts

have no available data (n=8)

No available data (n=24)

47 studies in Meta-analysis

(13 studies in English and 34 in Chinese)
