# Supplementary figures and images for: The Naturally Occurring YMDD Mutation among Patients Chronically Infected HBV and Untreated with Lamivudine: A Systematic Review and Meta-Analysis
Source: PLoS One. 2012 Mar 27;7(3):e32789. doi: 10.1371/journal.pone.0032789 (PMC3314000; doi:10.1371/journal.pone.0032789)

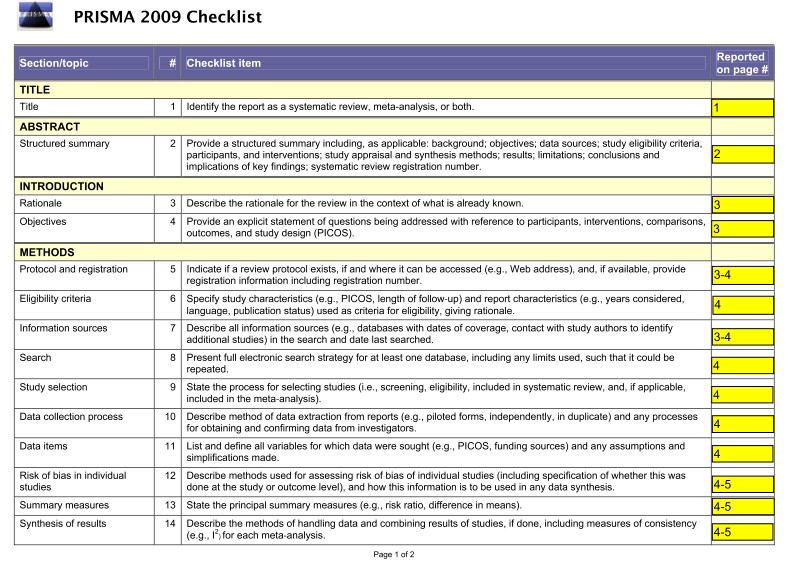


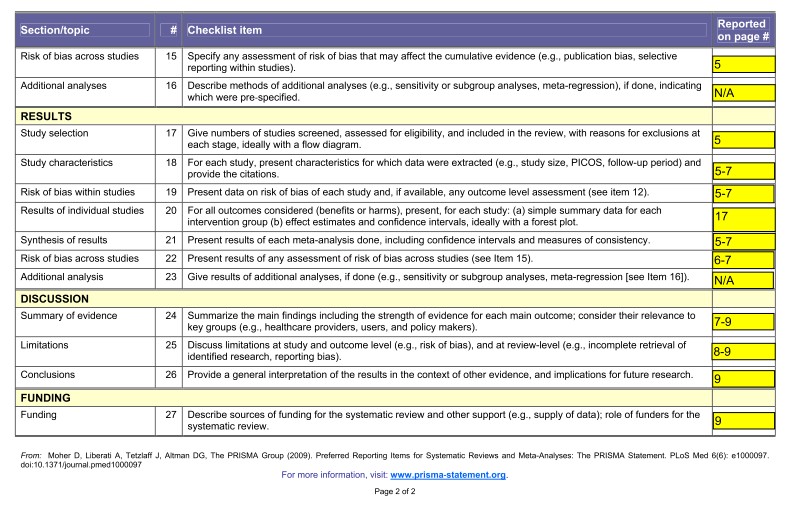

Supplement: Table S1 — PRISMA Checklist. (DOC) [file pone.0032789.s002.doc]
